# Spine Posture, Mobility, and Stability of Top Mobile Esports Athletes: A Case Series

**DOI:** 10.3390/biology11050737

**Published:** 2022-05-11

**Authors:** Wing-Kai Lam, Bob Chen, Rui-Tan Liu, James Chung-Wai Cheung, Duo Wai-Chi Wong

**Affiliations:** 1 Sports Information and External Affairs Centre, Hong Kong Sports Institute, Hong Kong 999077, China; gilbert.lam@connect.polyu.hk; 2 Dr Chen Sport Training and Rehabilitation Research Center, Beijing 101111, China; ruitan.liu@liningstar.com; 3 Department of Biomedical Engineering, Faculty of Engineering, The Hong Kong Polytechnic University, Hong Kong 999077, China; james.chungwai.cheung@polyu.edu.hk

**Keywords:** mobile game, sport injury, electronic sport, ergonomic, prolonged sitting, spine biomechanics, smartphone esports

## Abstract

**Simple Summary:**

Professional mobile esports athletes may suffer from occupational health risks to their spine because of prolonged sitting with poor posture. The aim of this study is to examine whether esports athletes have poor spine posture, mobility, and stability. Our results showed that professional mobile esports athletes had significantly poorer spine posture, and weaker spine mobility and stability than non-athletes. They may be more susceptible than non-athletes to spine-related musculoskeletal problems, such as back pain or neck pain. Physical-training programs and other ergonomic considerations should be sought to mitigate the risk of musculoskeletal problems and potential injuries for the athletes.

**Abstract:**

Professional esports athletes spend a long time in the same sitting posture during training and competition. Mobile esports may exacerbate potential postural problems because of the closer and unsupported arms and because athletes spend more time in a forward-/flexed-head posture. Prolonged sitting in these postures carries significant health risks and may lead to musculoskeletal problems and injuries. The objective of this retrospective study is to assess the posture, mobility, and stability of the spine for professional mobile esports athletes. We collected spine-assessment data from 48 athletes participating in a top-tier league on a real-time-strategy battle-arena online game. The spinal assessment was conducted using the SpinalMouse^®^ under upright standing and trunk flexion in addition to the Matthiass test. Measurements were converted into Idiag Scores by the SpinalMouse^®^ software. The Idiag Posture, Idiag Mobility, and Idiag Stability scores were 62.50 (IQR: 21), 63.50 (IQR: 19.5), and 54.50 (IQR: 14.5), respectively, and were significantly lower (*p* < 0.001) than the reference normative value (100). Age was found to have a weak positive correlation with the posture score (ρ = 0.29, *p* = 0.048). Although career duration appeared to lower the scores, the association was insignificant (*p* > 0.05). The scores also had no significant association with body height, body mass, body mass index, and esports team (*p* > 0.05). It was anticipated that mobile-based esports would attenuate the biomechanics of the spine and increase the likelihood of musculoskeletal problems, such as neck and back pain.

## 1. Introduction

Video games have become one of the greatest recreation industries, surpassing the music and movie industry [1]. In 2019, the revenue of the video-game industry was USD 152 billion, and that figure is expected to reach USD 257 billion by 2050 [1]. Many gameplayers are teenagers. More than one-fifth and one-third of gameplayers are aged below 18 and aged between 18 and 35, respectively [2]. In the United States, 90% of children regularly play video games, and 15% of children consider it their favorite media activity [3,4]. Meanwhile, the video game industry has evolved into a “sports” industry, termed “esports,” which refers to video-game competition [5,6]. Some esports competitions have a larger audience than major world leagues in traditional sports [7].

Nowadays, professional esports gameplayers are recognized as athletes who compete full-time as a career instead of playing video games as a hobby [8]. Elite esports athletes can earn sponsorships or prizes worth more than 1.5 billion dollars [9]. Colleges in the United States offer scholarships for students pursuing professional game competitions [10] and have set up relevant athletics departments [11,12], while universities in South Korea acknowledge esports as sporting events [13]. With its rising popularity, esports has made a debut in the 2022 Asian Games medals, and its sights are set on the 2024 Olympic Games [14,15]. Compared to general gameplayers, esports athletes require prolonged training for these significant scholarship and prizes.

However, esports athletes can suffer from both sports injuries and occupational health risks due to prolonged sitting in poor postures and repetitive movements. Athletes have reported numerous overuse injuries and repetitive strain injuries such as “Nintendonitis” and “Nintendo Neck” [1,16], as well as tennis elbow, leaguer’s shoulder, and gamer’s thumb. More than 40% of esports athletes have reported musculoskeletal pain [17] that could become tendinopathy [18]. In addition, esports activities were considered sedentary behavior [19] that manifested similar health risks to those of office workers with prolonged occupational sitting [20]. They spent 4–10 ho a day sitting statically in front of the screen, only performing fine hand–finger movements rigorously [21,22], and their occupational sitting is three times more physically demanding than that of typical office workers [23]. It was reported that about 40% of esports athletes did not participate in any other physical activities [3]. There was a high prevalence of hand pain, wrist pain, neck pain, and eye strain among esports athletes who received attention from health practitioners and esports coaches [3]. They attempted to mitigate this risk by incorporating physical activities/recovery measures during the training program for esports athletes [3,21]. The biomechanics analysis of esports maneuvers can provide a better understanding of the injury mechanism and facilitate ergonomic designs and configurations, such as seats, screens, and controllers [22,24,25]. 

While most of the esports research has been predominantly focused on the effects of computer-based games, little attention has been paid to mobile-gaming-based athletes. The transition from computer-based to mobile-gaming-based esports requires additional research to further understand and facilitate training designs that can mitigate occupational health risks. Mobile esports affects the posture of its athletes more than computer-based esports. Athletes struggle with a smaller screen, which leads to a forward- or flexed-head posture for better eye focus, while the elbow may dangle without a table or may press on the table with the athlete putting their body weight on their olecranons. Gameplay with handle-less controls demonstrate a narrower distance between arms, increase the shoulders’ internal rotation, and thus induce rounded shoulders and chest compression (angina pectoris). Prolonged aberrant forward-head posture, rounded shoulders, and chest compression lead to a posterior tilt that eventually causes back pain or spinal disorders. Existing studies report that neck and trunk pain appeared after 16 min of mobile gaming [26], while mobile-phone usage longer than two hours per day significantly increased the prevalence of neck, shoulder, and lower back pain among youths [27]. Therefore, it was anticipated that mobile esports athletes could also have a higher chance of spine-related musculoskeletal problems, such as neck and back pain. While one survey reported that 40% of mobile esports athletes complained of neck pain and a quarter of them complained of upper back pain, there was insufficient reporting on the prevalence of musculoskeletal injuries and their interventions in both computer-based and mobile-based esports [28]. 

The objective of this study is to investigate the posture, mobility, and stability of the spine among top esports athletes. We hypothesized that the spinal posture, mobility, and stability of the spine among top esports athletes would significantly deviate from the normative reference values. 

## 2. Materials and Methods

### 2.1. Participants

This study included the best starting-five athletes from each of the ten esports teams that joined a five-on-five mobile esports league in 2021 in Shanghai, China. The competition was a top-tier esports league named “Onmyoji Arena Pro League” that involved a multiplayer online game under the real-time-strategy battle arena (MOBA) genre. This was a retrospective case-series study followed by our other study about self-reported complaints and lifestyle on the same cohort [29].

Physical assessments were conducted for the participants. The data from 48 athletes were collected, since 2 of the 50 athletes did not complete the entire spinal assessment and declined re-assessment. We obtained verbal consent from participants for the retrospective use of their data for research. All participants were male. The mean age of the esports athletes was 20.1 years (SD: 1.67, range 18–24) with a professional career duration of 8.5 months (SD: 6.05, range 3–36). Their mean body height was 175.5 cm (SD: 5.83, range 167.0–193.0), while their mean body mass was 69.6 kg (SD: 16.5, range 47.5–118.0). Their mean body mass index (BMI) was 22.49 (SD: 4.85, range 16.0–36.4).

A post hoc statistical-power analysis was conducted using G*Power (ver. 3.1.9.4., Heinrich-Heine-University, Düsseldorf, Germany). We used a one-sample Wilcoxon Signed-rank test model (one-tailed) with a medium-effect size (d = 0.5), a significance level of 5%, and the recruited sample size (*n* = 48). We achieved a statistical power of 0.954.

The study was approved by the Institutional Review Board (Reference No.: HSEARS20220215002). All participants received information about the research and agreed to participate in the study.

### 2.2. Procedure

The spinal assessment was carried out using SpinalMouse^®^ (Idiag M360, Volkerswill, Switzerland). The device consisted of a wireless computer-mouse-like hand-held roller and scanned the spine by non-invasively gliding the device manually down the spinal column in different postures. The sampling frequency of the device was 150 Hz, and the device recorded data approximately every 1.3 mm. The experimental protocol, including the postures and the Matthiass test, was based on the assessment protocol of the SpinalMouse^®^ system. The device demonstrated good validity and reliability [30] and was applied previously in a spinal assessment for sedentary workers with non-specific lower back pain [31]. 

During the spinal assessment, the participants wore no clothes from the waist up, and they also wore no shoes. The levels of C7 and S3 were identified by palpation and marked. During each scanning condition, the experimenter placed and aligned the device at the C7 level spinous process according to the device instructions. Scanning procedures were carried out in three postures, namely, standing upright, performing trunk flexion, and the Matthiass test condition [32]. The participants balanced standing with their knee extended and their feet placed at hip width during the upright-standing posture. For the trunk-flexion posture, starting from upright standing, the participants slowly bent their trunk forward maximally with their knees extended, and their neck and upper limbs relaxed and leaned down. After that, the Matthiass test was conducted with participants starting from an upright standing posture and carrying a pair of dumbbells in accordance with individual body mass, as shown in Table 1 [33]. They lifted their arms to shoulder level, and the first set of measurements was taken. The participants then maintained their posture for 30 s for the second set of measurements. 

### 2.3. Data Reduction and Indicators

The data processing and derivation in the outcome indicators were conducted in the SpinalMouse^®^ software. The system calculated the intersegmental angles of the vertebra for all 17 segments (T1/T2 to L5/S1) by measuring the vertebra orientation in relation to the vertical axis perpendicular to the ground, and an intelligent recursive algorithm of the software processed the information [31]. 

The Idiag Posture, Mobility, and Stability scores were simplified indicators of the intersegmental angles in different postures to facilitate a quick assessment. The score indicators and internal specifications, including the dataset of reference normative range/value, were devised and benchmarked by the Company of the SpinalMouse^®^ (Idiag M360, Volkerswill, Switzerland). 

For the Idiag Posture score, each vertebral intersegment angle under the upright standing posture was compared to the range of reference normative values, which was provided by the software database based on the participant’s age and gender. The comparison for each intersegment was compiled into a point scale from 0 to 100. The point scale was then penalized by two harmony factors, including joint incongruency and flat curve (i.e., >7° in neighboring segments and <±1° in neighboring segments, except T12/L1). A higher score implied a greater agreement with the normal reference group, whereas a low score implied a lower degree of agreement. The Idiag Mobility and Stability scores were calculated based on the same principle, except that they considered the angle movement from upright standing to the flexion posture and the before–after measurements in the Matthiass test, respectively. 

### 2.4. Statistical Analysis

All statistical analyses and data visualization were conducted using R statistical package (Foundation of Statistical Computing, Vienna, Austria), and statistical significance was demonstrated when *p* < 0.05. All data were checked for outliers using the 1.5 interquartile range and normality using the Shapiro–Wilk test. 

Due to the violation of data normality or outlier assumption, a one-sample Wilcoxon Signed Rank test was used to test for significant differences between the Idiag indexes and corresponding normative values (i.e., point = 100) adjusted by Bonferroni correction. To evaluate the influence of potential confounding factors, the association of the scores with body height, mass, age, and year of competition was assessed with the Spearman’s test. In addition, the Kruskal–Wallis test was used to evaluate any confounding effects of team membership on the scores. 

## 3. Results

**Figure 1:**
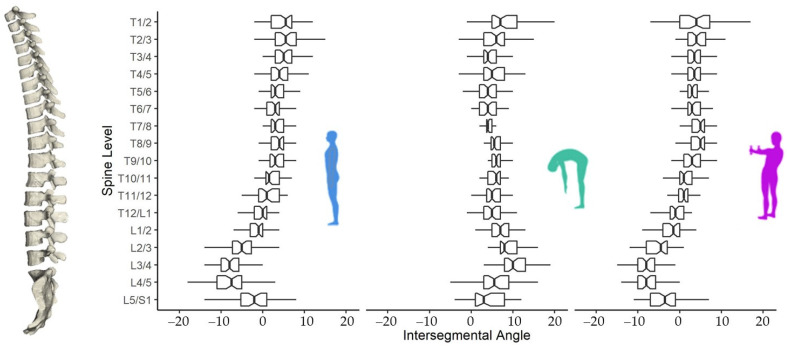
Intersegmental angles at different vertebral levels of the mobile esports athletes under balanced standing, trunk flexion, and Matthiass test.

**Figure 2:**
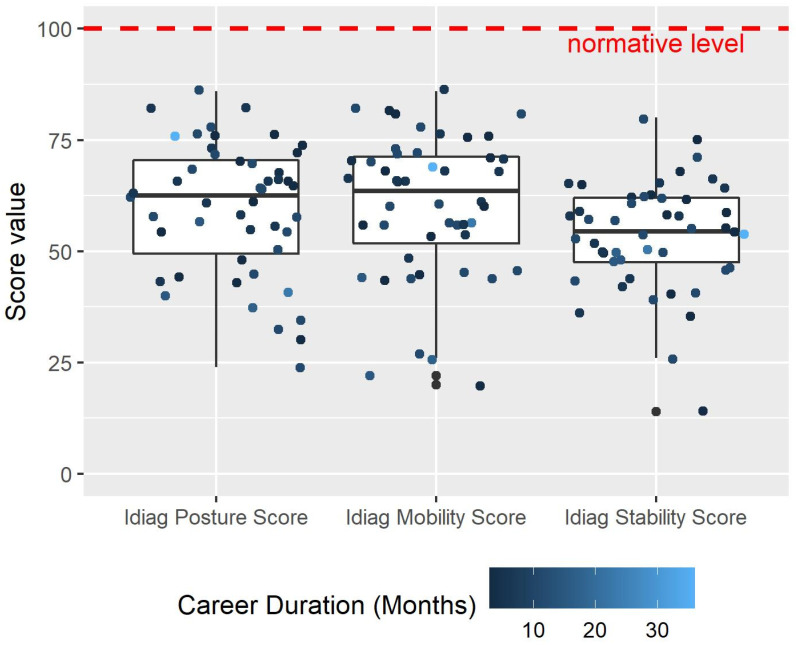
Idiag Posture, Mobility, and Stability scores of the participants. All scores show statistically significant difference from the reference normative level (*p* < 0.001).

Figure 1 shows the summary of the intersegmental angles in different postures, in which the raw data are available in the Supplementary Materials (Table S1). All Idiag scores were significantly different from the normative value. As shown in Figure 2, the median Idiag Posture score was 62.50 (Q1: 49.50, Q3: 70.50, *p* < 0.003), the median Idiag Mobility score was 63.50 (Q1: 51.75, Q3: 71.25, *p* < 0.001), and the median Idiag Stability score was 54.50 (Q1: 47.50, Q3: 62.00, *p* < 0.001). 

**Figure 3:**
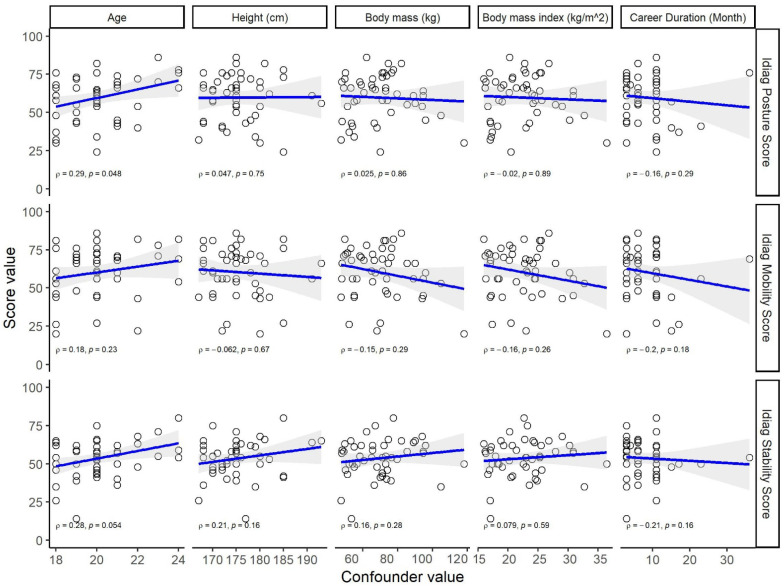
Association of Idiag scores with body height, body mass, and BMI.

As shown in Figure 3, there were no associations between the scores and height, mass, BMI, and career duration (*p* > 0.05). Age had a significant weak correlation with the posture score (ρ = 0.29, *p* = 0.048) and a marginally significant weak correlation with the stability score (ρ = 0.28, *p* = 0.054). There were 10 esports teams among the 48 participants. The teams of the athletes had no significant association with the Idiag Posture score (*p* = 0.892), Idiag Mobility score (*p* = 0.403), and Idiag Stability score (*p* = 0.314).

## 4. Discussion

To our best knowledge, this is the first study that evaluated the consequences on spinal biomechanics of top-tier mobile esports game athletes, while mobile esports were gaining popularity in relation to computer-based esports. Most existing research targeted case reports, cross-sectional studies that highlighted the incidence [1] and prevalence of health issues [3,34], or qualitative analysis from a public health perspective [19,22]; others focused on the computer-based esports category [22,23]. While prolonged occupational sitting was associated with chronic diseases and premature deaths [35,36], prolonged mobile posture (forward neck, slouched, and rounded shoulders) imposes additional health risks on the musculoskeletal system [37,38]. Mobile-game-based problems are more challenging and need to be corrected by improving the environmental ergonomics, such as using adjustable seats, displays, and tables, like that of the computer-based video game, and should have different considerations [39]. Regardless, both mobile-based and computer-based esports are calling for more extensive research towards better assessment and management [23].

This study provides substantial evidence of the harmful effects that professional mobile esports gaming has on athletes’ spines, and the impact of this study lies in its potential to inform practice for preventive training programs and therapeutic interventions. Our study found that mobile esports athletes had significantly worse spinal posture, mobility, and stability. However, body mass, height, BMI, career duration, and team membership were not correlated with the spinal assessment outcomes despite the fact that some of these factors were found to be associated with spinal curvature [40,41]. 

There is abundant evidence showing the negative impact of prolonged mobile usage in the general public, and such evidence bears a resemblance to the physical problems found in mobile esports athletes. More than two-thirds of mobile users reported musculoskeletal pain, including headache, neck, shoulder, upper extremity, and back pain [42,43,44,45], while the frequency of mobile gaming was associated with shoulder pain [46]. Existing studies revealed that the spine kinematics of mobile gaming posture were characterized by a forward-head or flexed-neck position, anterior trunk inclination, and increased thoracic kyphosis and lumbar lordosis, in which the increased exposure from these abnormal positions could lead to increased loads on extensor muscles [44,47]. 

Forward-head posture was the most problematic issue in mobile usage. Mobile-phone usage produced a notable forward-head posture, especially in a sitting posture without backrest [48]. Mobile-phone usage also demonstrated a higher level of neck flexion than other recreational activities, such as video-watching [49]. The prolonged abnormal head position required more contractive forces from relevant postural muscles for support [50,51]. Headache and neck pain were caused by the shortening of the posterior cervical extensor muscles and tightening of the anterior cervical muscles [52], which led to a kinetic chained effect on the upper trapezius, levator scapulae, and serratus anterior that induce abnormal scapula tilt, rounded shoulder, and thus shoulder pain [37,52,53]. 

In addition, mobile-gaming and –usage posture contributed to middle-back and lower-back pain [42,43,44,45]. The anterior trunk tilt accompanied by thoracic kyphosis increased the compressive and shear forces of the vertebrae and burdened the erector spinae and upper trapezius muscles [54,55,56], while lumbar lordosis increased the pressure of the intervertebral discs and spinal ligaments [57]. The low back pain could also be attributable to the compensation of increased pelvic tilt [54].

Compared to existing kinematics studies, ours is dedicated to understanding the long-term effect on the spine. The forward-head and abnormal-thoracic postures affected the range of motion and flexibility of the spinal column in the long run [58]. It shall be noted that some of the esports athletes were males whose ages are around puberty, in which the abnormal spine loading and characteristics may affect their normal growth. Our findings also found some relationships between age and spine-posture score.

There were some limitations in this study. We could not obtain the detailed calculation methods of the Idiag scores and detailed information on the normative reference dataset despite the fact that they were benchmarked and readily used in the clinical sector. We endeavored to enhance our data transparency by incorporating the raw intersegmental angle information in the manuscript. Moreover, the research was confined to a case-series that compared the outcome to a benchmark normative dataset to implicate the abnormality of spinal biomechanics. It shall be noted that smartphone users or ordinary mobile gamers with excessive screen-time also reported musculoskeletal pain and discomfort [59]. We could not make a distinction between the professional esports career and screen-time behavior in this study without a control group. In addition, we believed that career duration could be a potential dominant confounding factor because it represented the exposure of occupational risks. Although career duration appeared to have a reduction trend on the spinal score, our results did not demonstrate a significant association. While rehabilitation or physical training of participants may relieve some spinal problems, one of the reasons for the lack of association could be that the majority of our participants had a short career timespan, and we lacked participants with longer careers. It shall be noted that the career timespan for professional esports players was rather short, with the competitive ages being from 15 to 20 years and retirement starting at 26 years old [60]. Therefore, their pre-occupation or pre-professional gameplay timespan and behavior could also have influenced their spine conditions. To date, there is no clear definition on professional gameplayers, whereas amateurs could claim themselves playing “competitively” or “professionally” in online tournaments [28,61]. Future studies may consider classifying the level of players based on both daily gameplay time and tournament levels to better understand the impact on health. 

In addition, some athletes reported different levels and sites of pain and discomfort that were not considered in this study. Different esports teams may also incorporate different forms of training and recovery strategy, such as physical training, stretching, and physical therapy. Our study did not find any significant differences between the teams’ spine indexes. However, future studies with actigraphy and the deep learning approach shall consider the average time of gameplay training, physical training, and rest per day of the athletes. The data were sourced from one particular tournament on one particular esports game, so external validity may be compromised by the type/episode of the esports game. Other types of competitive games, such as card games, first-person-shooter games, fighting games, car-racing, and sports simulations, could have different paces, different play and rest time, stress, and levels of movements [62,63,64]. We selected the MOBA game in this study because it is believed to be the most outstanding achievement in contemporary esports events [65]. In terms of the experimental procedures, the spinal posture was estimated by scanning back contour using the spinal mouse that, in theory, may not process actual three-dimensional alignment and movement of the spine, though some studies attempted to use a similar approach via integrated accelerometers [66] or indirectly predicted the spinal alignment via body pressure measurement [67]. Ultrasound imaging can visualize the actual geometry and orientation of the posterior vertebral using both estimation [68,69] and volumetric rendering techniques [70]. This may serve as a better non-invasive alternative to the spinal mouse. At the same time, a tissue hemodynamics analysis via near-infrared spectroscopy could also help us understand the muscle-fatigue pattern during gameplay [71]. 

## 5. Conclusions

Mobile esports athletes demonstrated significantly poor spine posture, mobility, and stability than the normal reference. There was no evidence that career duration was associated with the spine posture, mobility, and stability, despite a declining trend. Age was found to have a weak but significant positive relationship with the posture score, while the other confounders were insignificant. 

## Figures and Tables

**Table 1:** Dumbbell mass carried during the Matthiass test.

Body Mass of Participant	Dumbbell Mass
<55 kg	2 × 1.5 kg
56–70 kg	2 × 2.0 kg
71–85 kg	2 × 2.5 kg
>86 kg	2 × 3.0 kg

## Data Availability

The authors confirm that the data supporting the findings of this study are available within the article and its Supplementary Materials.

## Supplementary Materials

The following supporting information can be downloaded at: https://www.mdpi.com/article/10.3390/biology11050737/s1, Table S1: Raw data on the demographic information and the intersegmental angle under different postures (Data ID = 40 and ID = 50 were missing data).
